# Learning of across- and within-task contingencies modulates partial-repetition costs in dual-tasking

**DOI:** 10.1007/s00426-021-01518-1

**Published:** 2021-04-22

**Authors:** Lasse Pelzer, Christoph Naefgen, Robert Gaschler, Hilde Haider

**Affiliations:** 1 Department of Psychology, University of Cologne, Richard-Strauss-Str. 2, 50931 Cologne, Germany; 2 Department of Psychology, FernUniversität in Hagen, Hagen, Germany

## Abstract

Dual-task costs might result from confusions on the task-set level as both tasks are not represented as distinct task-sets, but rather being integrated into a single task-set. This suggests that events in the two tasks are stored and retrieved together as an integrated memory episode. In a series of three experiments, we tested for such integrated task processing and whether it can be modulated by regularities between the stimuli of the two tasks (across-task contingencies) or by sequential regularities within one of the tasks (within-task contingencies). Building on the experimental approach of feature binding in action control, we tested whether the participants in a dual-tasking experiment will show partial-repetition costs: they should be slower when only the stimulus in one of the two tasks is repeated from Trial *n* − 1 to Trial *n* than when the stimuli in both tasks repeat. In all three experiments, the participants processed a visual-manual and an auditory-vocal tone-discrimination task which were always presented concurrently. In Experiment 1, we show that retrieval of Trial *n* − 1 episodes is stable across practice if the stimulus material is drawn randomly. Across-task contingencies (Experiment 2) and sequential regularities within a task (Experiment 3) can compete with *n* − 1-based retrieval leading to a reduction of partial-repetition costs with practice. Overall the results suggest that participants do not separate the processing of the two tasks, yet, within-task contingencies might reduce integrated task processing.

## Introduction

Whether it is cooking a meal, driving a car or being at work, in our everyday lives, many situations require us to perform tasks simultaneously. In most of these cases, performing two or more tasks comes along with performance costs. To investigate these dual-tasking costs, many researchers use the psychological refractory period (PRP) paradigm. In the original PRP paradigm (Pashler, 1994), the participants process two tasks within a trial which are presented with varying temporal overlap. The typical finding here is that the response times of the secondary task are slower, the higher the temporal overlap between the primary and the secondary task is (Pashler, 1994; Telford, 1931).

These dual-task costs are a robust phenomenon, but the debate about the sources of this processing limitation is still going on (for a current review, see Koch et al., 2018). Some of the dominant theories of dual-task performance assume structural properties, like a central bottleneck, in which central processing of the two tasks cannot run in parallel (Pashler, 1994), or central resource limitations inherent in the cognitive architecture, in which a central pool of resources has to be shared by both tasks (Kahneman, 1973; Navon & Miller, 2002; Tombu & Jolicoeur, 2003; Welford, 1952). Others suppose a strategic adaptation to circumvent task interferences, meaning a voluntary postponement of task processing by prioritizing one task over the other (Logan & Gordon, 2001; Meyer & Kieras, 1997). In short, one of the main question here is whether the two tasks can be processed only in a serial manner due to a structural bottleneck (Pashler, 1994), or whether parallel processing is possible but less efficient compared to serial processing (e.g., Logan & Gordon, 2001; Miller et al., 2009).

Despite this debate, these dual-tasking accounts share the central, albeit supposedly implicit assumption that both tasks are represented as two distinct task-sets. A usual way of thinking about dual-tasking is that the participants conduct two separate tasks. If both tasks overlap in their features, parallel processing leads to conflicts. To cope with these conflicts, the participants tend to process the two tasks in a serial manner. In turn, this serial processing then produces costs (cf. Fischer & Plessow, 2015; Logan & Gordon, 2001).

An alternative way of thinking about dual-task situations, however, is to assume that the participants might, at least when both tasks occur in temporal proximity, integrate the two tasks into one single task-set (Freedberg et al., 2014; Schumacher & Hazeltine, 2016; Künzell et al., 2018). Instructions in dual-tasking experiments usually tell the participants that each trial is composed of two tasks and that they have to respond to both of them. Additionally, feedback for both tasks is often administered after the secondary task’s response. With such instructions and feedback procedures two different ways of conceptualizing the two tasks appear plausible: either as one single task-set leading to an integrated representation of the two tasks or as two separate task-sets, one for each task.

Even though task integration is broadly discussed in the field of motor control (e.g., Mechsner et al., 2001; Summers et al., 1993; Swinnen & Wenderoth, 2004), there is surprisingly little research focusing on this question in cognitive research on dual-tasking (Schumacher & Hazeltine, 2016; Künzell et al., 2018; Liepelt et al., 2011; Neumann, 1984; Ruthruff et al., 2006; Schmidtke & Heuer, 1997; Zhao et al., 2020). The goal of the current study is to investigate such task integration and its role in dual-tasking.

## The role of task-sets

From task-switching and single-tasking studies, it is well known that the actual task-set plays an important role in how a task is processed. For instance, Dreisbach et al. (2007; see also Dreisbach & Haider, 2008, 2009) compared two conditions in a task-switching experiment. In the S–R condition, they told the participants the concrete stimulus–response mappings, while in the task-set condition, the participants received two different categorization rules. In both conditions, the participants then responded to the same eight stimuli during training. The results revealed task-switching costs in the task-set condition, but not in the S–R condition. Apparently, depending on the respective instruction, the participants generated an entirely different task representation.

To open the full scope of the influence of task-sets on task processing, in the field of implicit learning, for instance, Gaschler et al. (2012) instructed the participants in an implicit learning task to represent the responses either as colors or as response locations. While the participants learned a sequence of colors in the former condition, they learned a sequence of response locations in the latter (for similar results, see also Eberhardt et al., 2017). What these findings suggest is that instructions can shape via the task-set how the tasks in an experiment are represented. Therefore, an important point in dual-tasking might be to ask if it matters whether the participants represent the two task as one single or two separate task-sets.

## Task integration in dual-tasking

Given this important role of task-sets, it is a bit surprising that in dual-tasking, the role of task-sets has received little attention (but see Schumacher & Hazeltine, 2016; Künzell et al., 2018). In addition, it is contrary to dual-task research in motor cognition science where the focus on task-sets is rather common. For instance, in bimanual control studies, it has been shown that complex bimanual tasks can be performed easily when the performer represents the tasks as one single temporal stream (Mechsner et al., 2001; Summers et al., 1993). By contrast, it is almost impossible to successfully conduct these tasks concurrently when representing them as independent tasks.

In the field of cognitive dual-tasking, a few findings already suggest the importance of instructions to modulate the participants’ representation of the two tasks. Most of these experiments implemented the serial reaction-time task (SRTT) as one task and a random secondary task. In the SRTT, first introduced as such by Nissen and Bullemer (1987), the participants see four marked locations on the screen which are mapped to respective response keys. In each trial, a stimulus appears at one location on the screen after which an assigned response key has to be pressed as soon as possible. Unbeknownst to the participants, the marked screen locations follow a regular sequence. Even though implicit learning is generally a robust phenomenon (e.g., Reber, 1993), it is often found to be hampered in dual-tasking, especially if the stimuli in the other task are drawn randomly (Hsiao & Reber, 2001; Rah et al., 2000; Röttger et al., 2019, 2021; Schmidtke & Heuer, 1997; Schumacher & Schwarb, 2009; Zhao et al., 2020).

With regard to the role of task-sets, a study of Halvorson et al. (2013) is of particular importance. The authors used a derivative of the SRTT together with a secondary task with stimuli in random sequence. They instructed the participants to represent both tasks either as one single task-set or as two separate task-sets. Implicit learning effects were obtained when the participants were instructed to represent the tasks as two separate task-sets. In contrast, when they were instructed to conceptualize the two tasks as one single task-set, implicit learning was impaired. In a similar vein, Schumacher and Schwarb (2009) presented a visual-manual SRTT and an auditory-vocal random task. When they told the participants to prioritize the SRTT, implicit learning was found. When they instructed them to process the two tasks with equal priority, implicit learning was impaired. Although Schumacher and Schwarb (2009) interpret their findings in favor of parallel response selection processes resulting in a distraction of implicit learning in the latter case, the findings also fit the assumption that the instruction might have changed the underlying task-set. It is conceivable that, in the case of task prioritization, the participants might have generated two distinct task-sets, one for each task. When they were told to treat the tasks as equally important, they generated one single task-set for both tasks. This then led to an integrated representation of the two tasks and as such hampered, due to the randomness of the secondary task, implicit learning processes (Schmidtke & Heuer, 1997).

Two further studies are also worth mentioning within this context, since they provide further evidence for the crucial role of how the tasks in a dual-task paradigm are conceptualized. Freedberg et al. (2014) showed that when the participants were instructed to represent both tasks in a dual-tasking experiment as belonging together, the contingencies between the stimuli in the two tasks were learned. Likewise, Schumacher et al. (2018) compared in a dual-tasking study the performance of participants who had to respond either bimanually to both tasks or unimanually to only one of the two tasks. The demands in stimulus and response processing were held constant across conditions*.* Crucially, they additionally manipulated the instructions. In both response conditions, participants were told to represent the two stimuli either as one single task-set or as two separate task-sets. The results showed performance decrements when participants represented the two stimuli as belonging together but had to respond unimanually. In contrast, when they responded bimanually, performance was worse when the participants represented the tasks as two different task-sets. Both studies suggest that even in dual-tasking, the instructions matter with regard to how participants will represent the two tasks.

Beyond showing that the participants in dual-tasking might sometimes represent the two tasks as one single task-set, the findings by Freedberg et al. (2014) additionally hint at another important point. Even though the researchers used a visual-manual and an auditory-verbal task, it seems that the participants were able to integrate the two tasks into one single task-set. It seems as if task integration can occur independently of whether the two tasks share any features apart from temporal proximity. The latter assumption receives further support from two other studies that also tested for implicit learning under dual-tasking conditions (Röttger et al., 2019; Schmidtke & Heuer, 1997). In the study of Schmitdke and Heuer, the participants were trained with the SRTT and received a tone-discrimination task within the response–stimulus interval of the SRTT. The stimuli in the tone-discrimination task followed a regular sequence that was either correlated or uncorrelated with the SRTT sequence. The implicit learning effects were large in the former and reduced in the latter condition. Although the two tasks did not overlap in any stimulus or response features, they were integrated into one single-task stream enabling sequence learning with entirely correlated sequences of stimuli, but hampering learning with uncorrelated sequences. Röttger et al. (2019) replicated and extended these findings. In their last experiment, they trained the participants with the SRTT and a tone-discrimination task. Half of the SRTT positions were consistently paired with one specific tone, while the pairings between the SRTT positions and tones of the other half varied randomly. The findings revealed that implicit learning was preserved for the fixedly, but hampered for the variably paired SRTT–tone combinations. Again, this finding suggests that the participants integrate the stimuli of the two tasks before they then can learn something about the sequence built into the SRTT (see also Röttger et al., 2021).

To summarize, the findings so far suggest that dual-tasking does not necessarily mean that the two tasks are represented as distinct task-sets. The participants sometimes seem to represent them as a single task-set, leading to an integrated representation of the stimuli. In bimanual coordination (cf. Künzell et al., 2018; Mechsner et al., 2001) or when the two tasks in a dual-tasking experiment follow a correlated sequence (Schmidtke & Heuer, 1997), this seems to support performance. However, this integration into a single task-set also takes place when it impairs performance as has been shown for dual-tasking experiments with an inherent sequence in one, but not in the other task. Even when the participants are told to generate separate task-sets hindering task integration, there seems to be a rather strong tendency to only generate a single task-set for both tasks, at least when the tasks are presented in temporal proximity.

## Episodic memory retrieval as a potential mechanism for task integration

One possible way to account for the rather strong tendency of integrating the two tasks into a single task-set is to assume that processing two tasks in temporal proximity results in a common memory episode in the sense of Logan’s (1988) Instance Theory (for similar ideas, see Moeller & Frings, 2017; Zhao et al., 2020). According to Logan’s Instance Theory, a direct consequence of task processing is that the stimulus together with its response is stored as an instance or episode in long-term memory. An episode is thought to represent all information attended during task processing. The attention hypothesis, put forward by Logan and Etherton (1994), further states that the participants will integrate co-occurrences between task-relevant and task-irrelevant information when these are attended during task processing.

With regard to dual-tasking, it is thus conceivable that when the two stimuli appear more or less concurrently in a trial, they are represented as belonging to a single task-set. Since the temporal gap between the two tasks might be rather short, an integrated single memory episode results (rather than two distinct memory episodes). If, in the next trial, one or both stimuli of the two tasks are repeated, the memory episode from the preceding trial is re-activated. In the case of only one repeated stimulus, the retrieval of the Trial *n* − 1 episode can entail the activation of a response that does not match the current stimulus. Conflict between the retrieved response and the one activated based on the stimulus presented might contribute to the dual-tasking costs found in many experiments.

One way to diagnose such a confusion is to build a sequence into one of the two tasks, like in the SRTT dual-tasking experiments of, for instance, Röttger et al. (2019). The impaired implicit learning can be seen as an indicator of task-confusion due to integrated task processing. An alternative way, we pursue here, is to more directly test for the aftereffect of the stimulus combinations of the just processed trial (Trial *n* − 1) on the current trial (Trial *n*). If the stimuli of both tasks repeat, the task processing in the current trial should be facilitated. When only one stimulus reappears in the current trial, task processing should be slowed.

## The present study

The goal of the present study was to investigate whether the two tasks in a dual-tasking paradigm are stored together in one single memory episode that is retrieved whenever parts of the two tasks repeat from Trial *n* − 1 to Trial *n*. To this end, we build on the experimental approach of the work on feature binding in action control (Frings et al., 2007, 2020; Hommel, 1998). For instance, Hommel (1998) instructed the participants to prepare one task and, before producing its response, to respond to a second task. When the stimulus and response features repeated, the participants responded faster than when only either the stimulus or the response features repeated. Hommel (1998) interpreted these partial-repetition costs in favor of event files (Hommel et al., 2001). Event files are assumed to contain the current binding between stimulus and response features. However, some authors assume that these event files are thought to be rather short-lived and might not survive across trials (Moeller & Frings, 2017; but see, Dreisbach & Haider, 2008; Frings et al., 2020; Zhao et al., 2020). By contrast, memory episodes according to Logan (1988) are stored in long-term memory and thus should produce aftereffects even across trial boundaries.

For the question at hand, the important point of this research on feature binding is that the logic of partial-repetition costs offers a possibility to investigate the proposed episodic retrieval account in the context of dual-tasking. If the participants conceptualize the stimuli as belonging to one single task-set, they probably will store them together with the responses as a joint memory episode. This memory episode will be retrieved whenever, in the next trial, at least one of the two stimuli of the preceding trial reappears. That means, if both stimuli are repeated from Trial *n* − 1 to Trial *n* (i.e., a full repetition), the memory episode is consistent with the current stimulus combination, and thus, response times should be accelerated. If, however, only one of the two stimuli repeats (i.e., a partial repetition), the memory episode conflicts with the current combination of stimuli, leading to slower response times. In the case of no stimulus-overlap between Trial *n* − 1 and Trial *n* (i.e., a full switch), there is no Trial *n* − 1 memory episode that could be activated.

The goal of Experiment 1 was to test whether we would find such partial-repetition costs in the context of dual-tasking. In Experiment 2a, we went one step further: if memory episodes are an automatic result of task processing (Logan, 1988), changing the frequencies of particular combinations of the two task’s stimuli should change the frequencies of the acquired memory episodes which in turn should modulate the partial-repetition costs. When, for instance, Task 1 consists of a stimulus on the left side requiring a left-hand keypress and Task 2 is always a low-pitched tone, this frequent stimulus combination should, with practice, compete for retrieval with the memory episode from Trial *n* − 1 and as such might eclipse the aftereffect of the preceding trial. If this were the case, the partial-repetition costs should attenuate with practice. In an additional Experiment 2b, we tested whether the participants would actually learn the frequent stimulus combinations by replacing them in one block by completely random combinations. Finally, Experiment 3 aimed to investigate whether increasing the coherence within one of the two tasks would also modulate the partial-repetition costs. To this end, the stimuli in Task 1 followed a very easy-to-learn sequence, while the stimuli of Task 2 were again randomly presented.

In all experiments, we used a version of the dual-task paradigm by Röttger et al. (2019), consisting of a visual-manual SRTT-like task and an auditory-vocal tone-discrimination task. In each trial, both stimuli occurred simultaneously. In the visual-manual task, the participants had to respond with spatially mapped response keys to a cross which randomly flashed in one of three marked screen locations. In the auditory-vocal task, the participants had to verbally respond to a randomly presented high or low pitched tones. In contrast to the Röttger et al. (2019), however, we did not test for hampered implicit learning. Rather, we focused on partial-repetition costs. Therefore, with the exception of Experiment 3, the visual-manual SRTT never followed any sequence. In Experiments 2a and 2b, the pairing between the SRTT positions and the tones was fixed for two positions and variable for the third one. It is important to note that, contrary to the work in feature binding, these tasks did not overlap in their stimulus or response features. Thus, any obtained aftereffect from Trial *n* − 1 to Trial *n* can only result from the retrieved memory episode of the preceding trial (Logan & Etherton, 1994).

## General method

### Apparatus and stimuli

The experiments were controlled by a custom-written software (Lazarus/FreePascal, compiled for Microsoft Windows). Placeholders for the visual SRTT target (an uppercase “X”) were three horizontally aligned white squares on a light gray background (100 × 100 pixels, separated by gaps of also 100 pixels). They were displayed at the center of a TFT monitor (19 in.; 1280 × 1024 pixels) which was connected with a standard PC. In each trial, the SRTT target occurred for 100 ms in one of the three white squares and the participants had to press the respective spatially mapped response key (Y, X, C, on a German QWERTZ-keyboard).

Concurrently with the SRTT target, either a high tone (900 Hz) or low tone (300 Hz) was played for 56 ms. The participants had to respond verbally by saying “hoch” [high] if a high pitched tone sounded and “tief” [low] in the case of hearing a low pitched tone. The tone stimuli were integrated with the participants’ verbal response into a single wave-file per trial using a sound mixer (Behringer XENYX 302USB) as a bridge between headset and PC. After the experiment, the RTs of the vocal responses were analyzed with a custom-written program in Scilab. The correctness of the vocal responses was additionally manually controlled.

### Procedure

All participants were instructed step by step. They started with 20 practice trials with only the tone-discrimination task and another 20 practice trials with only the SRTT. They then received 20 practice trials with the dual-task setup. In this practice phase, all stimulus combinations were randomly presented. Immediately after the practice phase, the training began with six dual-task blocks of 108 trials each. Trials and stimulus combination within trials were presented in random order in Experiment 1. In Experiment 2, the order of the stimuli was also random, but two of the overall three combinations of stimuli in the SRTT and the tone-task were fixed, while the third was variable. In Experiment 3, an easy-to-learn sequence was implemented into the SRTT (1-2-3-3-2-1), while the tones appeared again randomly. In none of the experiments, the participants received any information about these regularities.

In all three experiments, a dual-task trial always began with the presentation of the visual SRTT target (the “X”) at one of the three different screen locations and, simultaneously, one of the two different auditory stimuli of the tone-discrimination task sounded. The participants were encouraged by instruction to give both tasks equal priority and to respond as fast and as accurately as possible. The response-window always closed 2000 ms after the onset of the stimuli and the next trial started immediately.

At the end of each experiment, the participant’s explicit knowledge about contingencies was assessed by asking them to verbally report any knowledge of contingency and to give a certainty judgment in percent. Participants who were able to report the correct contingencies were categorized as having explicit knowledge.

## Data analysis

Since our main goal was to investigate whether the participants would show partial-repetition costs as an aftereffect of the stimulus combinations in Trial *n* − 1 on Trial *n*, we focused our analyses on the differences between full repetitions (FR, both stimuli re-occur), full switch (FS, both stimuli change), and partial repetitions (PR, one stimulus changes) at the beginning (Block 1) and at the end of the training (Block 6). To this end, we conducted for each experiment linear interaction contrasts between the different trial types (FR, FS, vs. PR) and the first versus last training block separately for the SRTT and the tone-discrimination task. To assess the robustness of the results, we further computed Bayes factors with JASP (JASP Team, 2020), using the Bayesian *t* test framework proposed by Rouder et al. (2009).

According to the episodic retrieval account, we assumed that whenever one or both of the stimuli repeat from Trial *n* − 1 to Trial *n*, this might activate the memory episode of the preceding trial (*n* − 1). If this were the case, we should find slower responses for PR compared to FR/FS at the beginning of training in all three experiments. In Experiment 1, these RT costs should remain constant over practice, since all stimulus combinations were equally frequent and the participants could not learn anything. By contrast, if the higher frequency of particular stimulus combinations in Experiment 2 changes the distribution of the memory episodes, they should gain, with training, a higher probability to be activated in the current trial. That is, the memory episode of Trial *n* − 1 might not be sufficiently strong enough to further affect performance. Therefore, we expected to find the partial-repetition costs at the beginning but not at the end of training. Finally, in Experiment 3, we also expected the PR costs to vanish with practice, because the sequence implemented in the SRTT should increase the within-task coherence, hereby strengthening the probability to represent the two tasks as separate task-sets.

In all RT analyses, we excluded trials if an error had occurred in either the SRTT or the tone-discrimination task, or if the RTs were faster than 200 ms or slower than 1500 ms. In addition, the first trial of each block was eliminated, since it has no precursor. Furthermore, we decided to include only partial-repetition trials in which the tone repeated. The reason for excluding all partial-repetition trials in which the SRTT position repeated lay in the design of Experiment 2. Here, two of the three SRTT positions were always fixedly paired with one respective tone. Due to this manipulation, partial repetitions of the SRTT position could only occur for the variably paired SRTT position leading to very few trials in this category. Finally, we replaced the data set of participants who made more than 30% errors in at least one of the training blocks by that of a new participant.

## Experiment 1

Experiment 1 aimed to investigate partial-repetition costs in dual-tasking. For this purpose, all SRTT positions were randomly presented and randomly paired with the tones of the tone-discrimination task. If our considerations concerning episodic retrieval in dual-tasking were correct, we should find partial-repetition costs at the beginning and at the end of training.

## Method

### Participants

Twenty-six students (6 men, 20 women) of the University of Cologne (mean age 21.54 years, SD = 5.01) participated in the approximately 30 min-long experimental session for either monetary compensation or course credit.

### Apparatus and stimuli

Apparatus and stimuli were as described in the General Method. Task combinations within trials as well as across trials were randomized. In each of the six blocks, the trial distribution was 16.82% FR, 42.06% FS, 24.3% PR of the tone, and 16.82% PR of the SRTT position. As already mentioned, the last category was excluded from data analyses.

## Results and discussion

Trials in which the SRTT stimulus repeated and the tone did not (16.82%) could not be used for our analyses. Furthermore, we excluded 6.92% of the trials due to exclusion criteria.^1^ The data-set of one participant was replaced by a new one due to more than 30% incorrect responses in the tone-discrimination task.

### Performance in the SRTT and the tone-discrimination task

Table 1 presents the mean RTs and the error rates as a function of block and trial type in the SRTT and the tone-discrimination task. Participants became faster with practice. More importantly, the trial type affected the mean RTs.

**Table 1:** Mean RTs, error rates and SDs in the SRTT and the tone-discrimination task as a function of block and trial type in Experiment 1

Block	Full repetition				Full switch				Partial repetition			
	*M*	SD	%Error	SD_Error_	*M*	SD	%Error	SD_Error_	*M*	SD	%Error	SD_Error_
SRTT												
1	473.76	68.95	0.43	1.51	502.36	106.34	0.34	0.82	521.34	95.51	0.59	1.42
2	472.52	83.25	0.43	1.51	501.24	108.49	0.26	0.96	512.59	104.37	0.44	1.25
3	455.41	77.98	1.07	2.23	474.30	102.08	0.34	1.03	490.91	100.78	0.44	1.66
4	452.64	71.89	0.43	1.51	467.30	97.05	0.60	1.48	492.48	97.49	0.30	1.05
5	444.71	75.24	0.64	2.40	456.80	100.08	0.60	1.01	463.33	97.16	1.18	2.61
6	446.38	75.07	0.85	3.40	462.60	92.72	0.77	2.26	476.04	104.46	1.18	2.83
Tone-task												
1	700.74	102.54	1.07	3.15	732.43	140.33	1.71	2.54	753.18	121.82	2.22	4.37
2	690.84	113.25	0.85	2.04	728.82	141.73	1.54	2.33	759.13	134.19	2.07	3.48
3	672.42	107.14	0.64	1.81	702.07	133.02	1.11	1.57	720.38	129.72	2.66	3.89
4	664.20	112.36	0.64	1.81	688.90	131.57	1.11	2.01	725.34	136.13	2.37	2.89
5	633.40	113.42	0.43	1.51	665.10	128.35	1.54	2.50	682.21	139.01	1.63	2.91
6	659.33	107.08	0.85	2.58	678.90	128.43	2.48	3.63	708.16	139.80	2.51	2.65

**Fig. 1:**
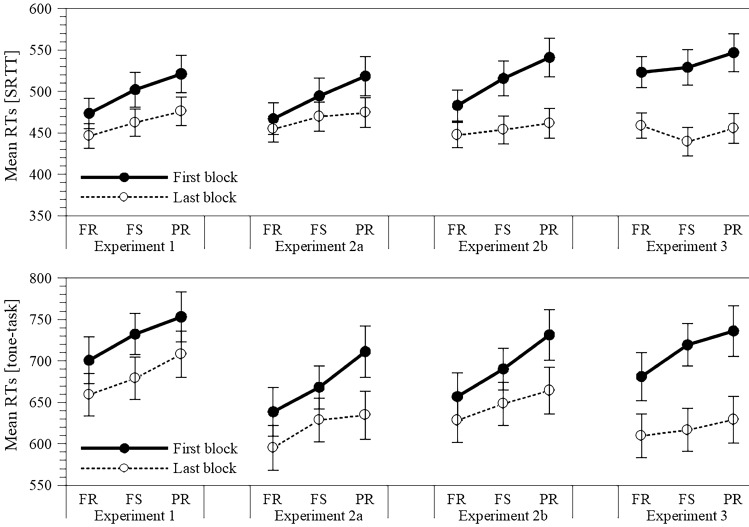
Mean RTs in the SRTT (upper panel) and the tone-discrimination task (lower panel) as a function of block (first and last block) and trial type (full repetitions, full switches, and partial repetitions) for each of the four experiments. Error bars represent standard errors

To provide a clearer picture of the effect of trial type on the mean RTs in the two tasks, Fig. 1 depicts these mean RTs as a function of trial type in Blocks 1 and 6. The figure shows that in both, the SRTT and the tone-discrimination task, the RTs for full repetition were faster than for partial repetitions.^2^ With practice, this RT increase (across the conditions FR, FS, PR) was slightly reduced in the SRTT, while it remained stable in the tone-discrimination task.

As described in the General Method, we computed the interaction contrasts comparing the linear trends in Blocks 1 vs. 6 separately for the SRTT and the tone-discrimination task. For the mean RTs in the SRTT, this interaction contrast just failed the level of significance, *t*(25) = 1.76, *p* = 0.08, *d* = 0.34. In the tone-discrimination task, there was no significant linear interaction contrast, *t*(25) = 0.3, *p* = 0.76, *d* = 0.06. For the error rates, the interaction contrasts yielded no significant differences, neither in the SRTT, *t*(25) = 0.293, *p* = 0.77, *d* = 0.06, nor in the tone-discrimination task, *t*(25) = 0.589, *p* = 0.56, *d* = 0.12.

We further conducted Bayesian paired sample *t* tests for the RTs and error rates by testing the null hypothesis (H_0_), that there is no difference in the linear trends between the first and the last block. We selected the default prior option, that is, a Cauchy distribution with spread *r* set to 0.707 (Jeffreys, 1961; Rouder et al., 2009). For the RTs, the Bayes factors indicate inconclusive evidence for the H_0_ in the SRTT, specifically, BF_01_ = 1.27, but moderate evidence in the tone-discrimination task BF_01_ = 4.63. For the error rates in both tasks, the SRTT, BF_01_ = 4.64, and the tone-discrimination, BF_01_ = 4.12, task Bayes factors yielded moderate evidence for the H_0_.

Together, the results of Experiment 1 showed two important results with regard to partial-repetition costs in dual-tasking. First, if the two stimuli repeat from Trial *n* − 1 to Trial *n*, the participants are significantly faster than when only the stimulus of the tone-discrimination task repeats, but the SRTT stimulus does not. This was the case for the SRTT and the tone-task as well. Second, the extent of these partial-repetition costs did not change much across training. This suggests that partial-repetition costs occurred in a dual-tasking paradigm. From a more methodological point of view, the findings thus show that our design is well suited to investigate the question whether increasing the frequency of particular stimulus combinations of the two tasks will modulate the partial-repetition costs.

## Experiments 2a and 2b

On the basis of these findings, we can now turn to the question whether a change in the frequencies of particular stimulus combinations will modulate the partial-repetition costs. For this purpose, the SRTT positions 1 and 2 were fixedly paired with one respective tone, while SRTT position 3 was randomly combined with either the high tone or the low tone. As in Experiment 1, order of the stimulus pairings within blocks was completely random. We expected that the unequal frequency distribution of stimulus combinations will, with practice, eclipse the partial-repetition costs. In Experiment 2a, we only tested whether the partial-repetition costs will vanish with practice. Experiment 2b was identical with the only exception that we additionally controlled for learning of the fixed task combinations. To this end, we replaced the fixed SRTT–tone combinations by random combinations in Block 5 and re-introduced them in Block 6.

## Method

### Participants

Forty-nine students of the University of Cologne participated in the experiment either for monetary compensation or course credit. 24 participants (8 men, 16 women; mean age 24.33, SD = 5.78) took part in Experiment 2a and 25 participants (12 men, 13 women; mean age 22.16, SD = 2.57) were in Experiment 2b.

### Apparatus and stimuli

Apparatus and stimuli were the same as described in the General Method. The only difference concerned the stimulus combinations in the training blocks. In Experiment 2a, the SRTT position 1 was always presented together with the low pitched tone and the SRTT position 2 with the high pitched tone. The SRTT position 3 appeared unpredictably with either the high tone or the low tone. In Experiment 2b, the participants received the same pairings as in Experiment 2a. However, in Block 5, all SRTT–tone pairings were random.

In all blocks, but Block 5 of Experiment 2b, the trial distribution was 28.04% FR, 42.06% FS, 24.3% PR of the tone and 5.61% PR of the SRTT position. In the random Block 5 of Experiment 2b, the frequencies were identical to those of Experiment 1 (16.82% FR, 42.06% FS, 24.3% PR of the tone, and 16.82% PR of the SRTT position).

## Results and discussion

In Experiments 2a and 2b of the last trial type, the PR of the SRTT (5.61% in Experiment 2a and 7.4% in Experiment 2b), was again excluded from further data analyses. Furthermore, due to our criteria, we excluded 3.79% and 6.1% of the trials in Experiment 2a and 2b, respectively.^3^ In addition, in Experiment 2a, the data of two participants had to be replaced due to too many unclassifiable vocal responses and due to more than 30% errors in the SRTT. In Experiment 2b, the data set of one participant was lost due to technical problems and was replaced by a new one.

### Performance in the SRTT and the tone-discrimination task

Table 2 contains the mean RTs and the error rates as a function of block and trial type separately for the SRTT and the tone-discrimination task of Experiment 2a. Table 3 shows the same for Experiment 2b. As can be seen, with practice, the participants in both experiments became faster and tend to make fewer errors. More importantly, the respective trial types influenced the mean RTs in both tasks. As in Experiment 1, responses were faster for full repetitions than for partial repetitions. However, here, these RT differences reduce across training.

**Table 2:** Mean RTs, error rates and SDs in the SRTT and the tone-discrimination task as a function of block and trial type in Experiment 2a

Block	Full repetition				Full switch				Partial Repetition			
	*M*	SD	%Error	SD_Error_	*M*	SD	%Error	SD_Error_	*M*	SD	%Error	SD_Error_
SRTT												
1	467.30	75.96	0.42	1.13	494.74	109.64	0.28	0.75	518.63	106.95	0.64	1.46
2	463.43	81.55	0.28	0.94	487.46	109.93	0.28	0.75	501.64	103.94	1.28	2.17
3	464.67	91.20	0.69	1.70	472.01	105.15	0.93	2.06	514.35	110.15	0.96	2.04
4	446.72	77.03	0.42	2.04	468.77	111.61	0.37	0.85	485.34	106.58	1.12	2.12
5	458.93	74.32	0.69	1.70	463.05	97.53	0.19	0.63	487.96	107.01	1.28	2.17
6	454.53	80.25	0.14	0.68	469.73	101.20	0.37	1.42	474.49	88.51	0.96	1.70
Tone-task												
1	638.70	107.65	1.11	2.12	668.31	142.17	1.39	1.83	711.27	144.50	2.88	4.85
2	612.31	116.08	0.00	0.00	647.75	143.54	1.02	1.60	665.37	137.93	1.44	2.73
3	605.50	115.92	0.83	2.03	631.58	140.35	0.93	1.72	670.86	149.28	2.08	4.39
4	578.10	96.35	0.42	1.49	607.05	142.66	1.11	2.86	633.64	129.64	1.76	2.77
5	581.66	111.93	0.42	1.13	600.93	131.61	0.65	1.39	622.68	136.82	0.80	1.60
6	595.28	114.65	0.28	0.94	628.84	144.95	0.56	1.18	634.61	122.11	1.28	2.17

**Table 3:** Mean RTs, error rates and SDs in the SRTT and the tone-discrimination task as a function of block and trial type in Experiment 2b

Block	Full repetition				Full switch				Partial repetition			
	*M*	SD	%Error	SD_Error_	*M*	SD	%Error	SD_Error_	*M*	SD	%Error	SD_Error_
SRTT												
1	483.24	84.61	0.13	0.67	515.87	113.49	0.18	0.62	541.13	107.44	0.77	1.57
2	463.93	81.74	0.67	1.36	489.24	115.44	0.36	0.83	513.76	121.90	0.62	1.44
3	459.11	71.07	0.13	0.67	474.14	104.39	0.44	0.91	502.79	99.94	0.46	1.28
4	452.59	74.08	0.40	1.47	468.53	90.09	0.44	1.11	479.97	99.38	1.08	1.76
5	463.21	70.77	0.89	2.08	489.42	97.11	0.36	0.83	493.30	96.99	0.31	1.06
6	447.51	68.35	0.53	1.25	453.92	84.70	0.44	0.91	461.81	92.88	0.46	1.28
Tone-task												
1	657.00	113.06	0.53	1.58	690.37	144.29	1.51	2.10	731.54	128.24	1.85	4.02
2	658.67	108.35	0.53	2.08	683.38	151.54	2.04	3.33	717.46	141.08	3.69	5.15
3	641.52	108.97	0.67	3.33	664.96	149.33	2.04	3.51	709.22	148.48	2.15	3.86
4	624.59	107.67	0.93	2.26	658.42	134.91	1.07	1.94	693.69	126.17	1.23	2.14
5	646.92	100.72	0.67	2.44	685.04	134.58	2.58	5.51	699.23	128.33	2.00	5.78
6	628.33	104.24	1.07	2.84	648.42	125.20	2.22	5.56	664.28	119.24	1.69	4.85

Figure 1 presents a clearer picture of the effect of the trial type on the mean RTs in Block 1 versus Block 6 for both tasks. In either task, the RTs in Block 1 increased almost linearly from the FR to the PR trial type. In Block 6, this increase was shallower. This pattern is obtained for both experiments.

The interaction contrasts comparing the linear trends of trial type in Block 1 vs. 6 in the SRTT confirmed this impression for the mean RTs. For both experiments, the interaction contrasts were significant for the SRTT, *t*(23) = 2.95, *p* < 0.001, *d* = 0.60, in Experiment 2a and, *t*(24) = 4.19, *p* < 0.001, *d* = 0.83, in Experiment 2b. For the tone-discrimination task the picture was quite similar: *t*(23) = 2.65, *p* < 0.001, *d* = 0.54 and t(24) = 3.15, *p* < 0.01, *d* = 0.63, in Experiments 2a and 2b, respectively. The error rates were overall rather low and did not differ between the three trial types. The interaction contrasts yielded no significant differences in the SRTT, neither for Experiment 2a, *t*(23) = 1.05, *p* = 0.305, *d* = 0.21, nor for Experiment 2b, *t*(24) = 1.51, *p* = 0.14, *d* = 0.30. This was also found in the tone-discrimination task in Experiment 2a, *t*(23) = 1.71, *p* = 0.10, *d* = 0.35, and Experiment 2b, *t*(24) = 0.97, *p* = 0.34, *d* = 0.19.

Again, we conducted also Bayesian paired sample *t* tests separately for the RTs and error rates. Here, we tested the two-sided alternative hypothesis (H_1_), postulating a difference in the partial-repetition costs between the first and the last block. The default prior option was again set to a Cauchy distribution with spread *r* set to 0.707 (Jeffreys, 1961; Rouder et al. 2009). For the RTs in the SRTT, the Bayes factors indicate moderate evidence for H_1_ in Experiment 2a, BF_10_ = 6.43, and strong one in Experiment 2b, BF_10_ = 92.71. For the RTs in the tone-discrimination task, the Bayes factor also indicate moderate evidence for H_1_ in Experiment 2a, BF_10_ = 3.62, and strong evidence for H_1_ in Experiment 2b, BF_10_ = 9.64. For the error rates, we found no evidence for H_1_, neither in the SRTT nor in the tone-task of both experiments (Bayes factors were BF_10_ = 0.35 and BF_10_ = 0.58 in the SRTT and BF_10_ = 0.76 and BF_10_ = 0.32 in the tone-task for the Experiments 2a and 2b, respectively).

**Fig. 2:**
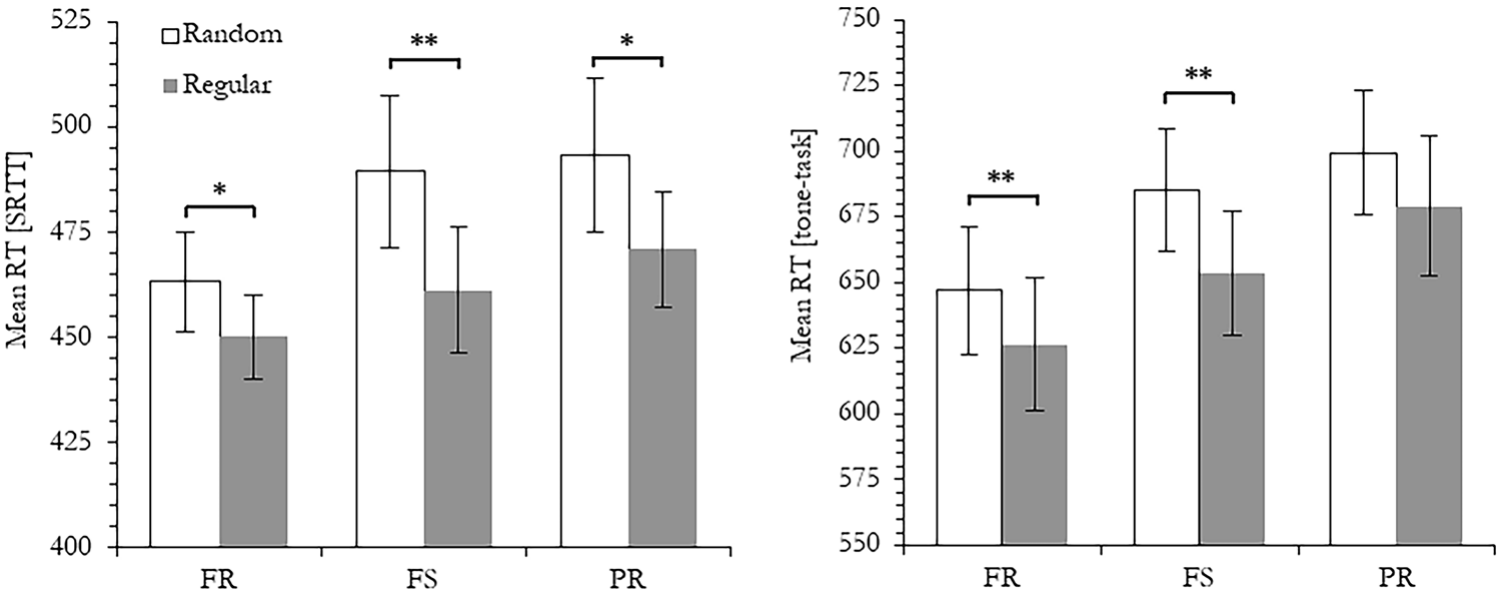
Sequence learning effects in the SRTT (left-side panel) and the tone-discrimination task (right-side panel) shown as differences in the mean RTs between the pooled regular Blocks 4 and 6 and the randomly paired tasks in Block 5, separately for each trial type (full repetitions, full switches and partial repetitions). Error bars represent standard errors. A significant difference in the *t* test is labeled with **p < 0.01 and *p < 0.05, respectively

In addition, we also analyzed whether the replacement of the fixed SRTT–tone combinations in Block 5 of Experiment 2b led to an increase in RTs in the two tasks. The *t* tests for the differences in the mean RTs between the pooled regular Blocks 4 and 6 and the randomly paired tasks in Block 5 were both significant (for the SRTT, the difference was 28 ms, *t*(24) = 5.15,* p* < 0.001, *d* = 1.03; for the tone-discrimination task, it was 31 ms, *t*(24) = 3.73, *p* < 0.01, *d* = 0.75). This suggests that the pairings between the SRTT-positions and the tones had been learned. As can be seen in Fig. 2, the RTs were prolonged for all three trial types, either in the SRTT or the tone-task. Separately computed *t* tests for each of these trial types yielded significant learning effects except for the PRs in the tone-discrimination-task (*t*(24) = 1.89,* p* = 0.07, *d* = 0.38).

For the error rates, the comparison between Block 5 and the two adjacent Blocks 4 and 6 did not indicate any significant difference, neither in the SRTT, *t*(24) = 0.24, *p* = 0.82, *d* = 0.05, nor in the tone-discrimination task *t*(24) = 0.99, *p* = 0.33, *d* = 0.2.

The additional Bayesian paired sample *t* tests confirmed the results regarding the effect of the replacement of the fixed SRTT–tone combinations in Block 5 (Jeffreys, 1961; Rouder et al., 2009). For the RTs, the Bayes factors revealed very strong evidence for H_1_ in both tasks (SRTT: BF_10_ = 835.87; tone-discrimination task: BF_10_ = 33.348). For the error rates, the Bayes factors indicated no evidence for H_1_ (BF_10_ = 0.22 in the SRTT, and BF_10_ = 0.33 in the tone-task).

Taken together, the findings of Experiments 2a and 2b once again suggest that it matters in dual-tasking whether the stimulus combinations are repeated from Trial *n* − 1 to Trial *n*. At the beginning of the training, the participants responded faster when both stimuli were repeated from Trial *n* − 1 to Trial *n* than when only the stimulus in the tone-discrimination task repeated. As hypothesized, Experiments 2a and 2b revealed additionally that presenting fixed SRTT–tone combinations modulated these partial-repetition costs. At the end of training, the linear increase of the RTs as a function of trial type was significantly reduced. Furthermore, the results of the Experiment 2b suggest that the reduction of this aftereffect from Trial *n* − 1 to Trial *n* was indeed due to the learning of the fixedly presented SRTT–tone combinations.

Overall, the findings of Experiments 1 and 2 are in line with our episodic retrieval account, proposing that the participants do not represent the two tasks as separate task-sets, but rather store them in joint memory episodes. If particular stimulus combinations appear more frequently, these frequent episodes eclipse the memory-retrieval effects derived from the preceding trial.

## Experiment 3

The last goal was to examine whether strong expectancies within a task also compete with the retrieval of the most recent memory episode jointly representing elements from both tasks. To this end, we implemented a simple and easy-to-learn sequence in the SRTT (1-2-3-3-2-1, positions from left to right). If the assumption holds true that increasing the coherence within one task can overwrite the aftereffects of Trial *n* − 1 on Trial *n*, we should find again a reduction in the partial-repetition costs from Block 1 to Block 6.

## Method

### Participants

Twenty-five students (6 men, 19 women) of the University of Cologne (mean age 23.44 years, SD = 3.57 years) participated in the experiment either for monetary compensation or course credit.

### Apparatus and stimuli

Apparatus and stimuli were the same as described in the General Method. The only exception was that now the SRTT positions followed a rather easy sequence (1-2-3-3-2-1). The stimulus combinations between the SRTT and the tone-discrimination task were random. In each of the six blocks, the trial distribution was similar to that of Experiment 1 with 16.82% FR, 42.06% FS, 24.3% PR of the tone, and 16.82% PR of the SRTT-position.

## Results and discussion

Again, trials in which the SRTT stimulus repeated and the tone did not (16.82%) were not used for our analyses. Furthermore, we excluded 12.67% of the trials due to exclusion criteria.^4^ Furthermore, we replaced the data of two participants as they had made more than 30% errors in each of the two tasks in the SRTT and in the tone-discrimination task.

### Performance in the SRTT and the tone-discrimination task

The mean RTs and the error rates as a function of block and trial type are presented in Table 4 for the SRTT and the tone-discrimination task. As can be seen from this table, the participants became faster and tended to make fewer errors with practice. Figure 1a and 1b depicts the mean RTs as a function of trial type in Block 1 and Block 6 for the SRTT and the tone-task. The picture resembles that of Experiment 2. Again, the mean RTs at the beginning of training are influenced by trial type. The participants responded slower when only one stimulus repeated than when both repeated. At the end of training, this linear increase of the mean RTs almost vanished.

**Table 4:** Mean RTs, error rates and SDs in the SRTT and the tone-discrimination task as a function of block and trial type in Experiment 3

Block	Full repetition				Full switch				Partial repetition			
	*M*	SD	%Error	SD_Error_	*M*	SD	%Error	SD_Error_	*M*	SD	%Error	SD_Error_
SRTT												
1	523.36	89.08	0.44	1.54	529.13	122.73	1.40	2.87	546.91	112.40	2.53	3.94
2	511.90	150.86	0.67	2.44	495.68	119.34	1.49	2.88	520.85	113.21	2.81	2.89
3	488.42	106.13	1.12	2.80	476.90	127.25	1.50	2.08	503.16	139.04	1.54	2.22
4	475.30	132.28	1.33	2.42	457.20	116.23	1.41	2.73	476.63	107.18	2.31	4.15
5	471.41	114.64	0.69	1.92	447.61	118.21	1.23	2.12	475.12	129.83	2.94	3.20
6	458.80	116.70	0.92	2.73	439.42	118.39	1.92	2.69	455.61	110.70	1.40	1.91
Tone-task												
1	681.24	145.46	1.12	2.80	719.54	178.03	3.25	3.34	736.15	168.51	4.23	6.67
2	655.00	139.62	1.35	2.92	652.40	171.16	3.61	4.55	698.54	170.05	2.95	4.79
3	624.25	145.73	0.89	2.63	652.20	169.87	2.55	2.89	669.36	178.80	2.31	4.30
4	617.81	150.03	1.11	2.27	628.28	174.47	2.46	3.76	651.48	157.25	2.01	3.19
5	613.49	141.25	1.58	3.05	625.06	159.59	2.37	3.65	661.51	159.92	2.50	5.27
6	609.62	139.14	0.68	2.47	616.69	163.93	2.63	3.41	629.23	147.27	2.19	4.58

We conducted again the linear interaction contrasts (Trial type X Block 1 vs. 6) with RT as the dependent variable separately for the SRTT and the tone-discrimination task. For the mean RTs, these linear interaction contrasts were significant in both tasks [*t*(24) = 2.57, *p* < 0.01, *d* = 0.51, for the SRTT, and *t*(24) = 2.88, *p* < 0.01,* d* = 0.58, for the tone-discrimination task]. This suggests that increased within-task coherence modulated the partial-repetition costs with practice. Like in the preceding experiments, the interaction contrasts for the error rates yielded no significant difference in the tone-discrimination task, *t*(24) = 1.2, *p* = 0.24, *d* = 0.24. In the SRTT, the interaction contrast just failed the level of significance, *t*(24) = 1.9, *p* = 0.07, *d* = 0.38.

The Bayesian paired sample *t* tests computed separately for the two tasks revealed for the RTs moderate evidence for the H_1_ in the SRTT, BF_10_ = 3.08, and in the tone-discrimination task, BF_10_ = 5.60. For the error rates, the Bayes factors again indicate no difference for both the SRTT, BF_10_ = 0.22, and the tone-discrimination task, BF_10_ = 0.33. Overall 12 participants could verbalize the sequence and were categorized as having explicit knowledge. Both for the SRTT and the tone-task, there were no significant differences between the participants who had explicit knowledge and those who had none when comparing the reduction in the partial-repetition costs from Block 1 to Block 6 [SRTT: *t*(23) = 0.46, *p* = 0.65, *d* = 0.09, tone-task: *t*(23) = 0.62, *p* = 0.54, *d* = 0.12].

Thus, the picture of the results is rather similar to that obtained for Experiments 2a and 2b. The partial-repetition costs are found at the beginning of training and then disappear with ongoing training. Yet, one limitation concerning the findings of Experiment 3 is that the reduction of the partial-repetition costs in Experiment 3 could have been caused by advance preparation. The easy-to-learn sequence used here might have allowed the participants to prepare the responses in advance of a trial. This should also reduce the partial-repetition costs with training. In a similar vein, sequence knowledge in single-tasking studies has been shown to increase performance in tasks in which control demands originate from added irrelevant distracting information, reducing the Stroop effect (Haider et al., 2011), the Simon effect (Koch, 2007; Tubau & López-Moliner, 2004), and the impact of biased transition frequencies (Tubau et al., 2007; Tubau, & López-Moliner, 2004). We will come back to this point in the General Discussion.

## General discussion

Several findings in the field of dual-tasking suggest that the participants tend to integrate the two tasks within a trial into one single task-set when the stimuli of these tasks are presented in temporal proximity (Schumacher & Hazeltine, 2016; Röttger et al., 2019, 2021; Schmidtke & Heuer, 1997; Zhao et al., 2020). The goal of the current study was to examine whether the generation of joint memory episodes might be a conceivable mechanism underlying task integration (Logan, 1988). The rationale was that the short temporal gap between the simultaneously presented stimuli might be insufficient to store them separately. Instead, a common memory episode of the stimuli and responses of both tasks in a trial might result. To investigate this question, we built on the logic of partial-repetition costs known from feature binding in action control (Hommel, 1998). The central assumption underlying partial-repetition costs is that if two successively processed tasks overlap only partially in their stimulus or response features, the event file generated to process the first task needs to be disintegrated before the second task can be processed (Hommel, 2004; Hommel & Frings, 2020). This leads to slower response times compared to cases in which all features repeat in the two tasks.

For two reasons, the partial-repetition logic in action control cannot be transferred directly to our research question. First, event files are thought to be rather short-lived, and second, the partial-repetition costs depend on feature-code overlap between tasks. By contrast, task integration in dual-tasking presupposes survival across trial boundaries (e.g., Moeller & Frings, 2017; Zhao et al., 2020), and seems to occur even when the tasks consist of a visual-manual and an auditory-vocal task which do not overlap in any feature code (Röttger et al., 2019, 2021; Schumacher & Schwarb, 2009).

To account for the first difference, we referred, in line with other researchers (Frings et al., 2020; Zhao et al., 2020), to Logan’s Instance theory instead of relying on event files. According to the Instance Theory (Logan, 1988), task processing leads to memory episodes which are stored in long-term memory. They are thought to represent all information which had been attended during task processing (Logan & Etherton, 1994). With regard to the second point, we argued for a crucial role of task-sets in dual-tasking (Dreisbach & Haider, 2009). It is by no means clear whether the participants represent the two tasks in a dual-tasking trial as different task-sets. Rather, a few findings suggest already that they generate a common task-set for the entire dual-tasking trial, at least, when no further instruction is given and the stimuli are presented simultaneously (Halvorson et al., 2013; Schmidtke & Heuer, 1997; Schumacher & Schwarb, 2009; Zhao et al., 2020). Based on these considerations, we hypothesized that partial-repetition costs should be obtained also in dual-tasking. That means, we expected to find, analogous to the partial-repetition costs in feature binding, slower RTs when only the stimulus of one task repeats from Trial *n* − 1 to Trial *n* than when the stimuli of both tasks repeat.

The results of the current experiments are rather clear-cut with regard to this hypothesis. In Experiment 1, the stimuli of the two tasks as well as the trials were randomly paired and occurred in random sequence, as is the usual case in dual-tasking. We found, for both tasks, the SRTT and the tone-discrimination task, slower RTs for partial repetitions than for full repetitions. In addition, these partial-repetition costs remained rather stable across training. In Experiments 2a and 2b, we manipulated the frequencies of particular SRTT–tone combinations. The findings replicated the partial-repetition costs at the beginning of training. However, here, the influence of the partial-repetition costs decreased over practice and was significantly smaller at the end of training. In addition, in Experiment 2b, we could show that replacing the frequent SRTT-tone pairings by random stimulus combinations decelerated the RTs. This indicates that the particular stimulus combinations were indeed learned. In Experiment 3, we went one step further and tested whether the within-task coherence of an implemented easy-to-learn SRTT sequence would affect the partial-repetition costs. The findings confirmed this assumption. Again the partial-repetition costs decreased significantly over training.

Thus, overall our current results suggest that partial-repetition costs occur in dual-tasking with non-overlapping task features. Furthermore, they show that whenever particular events are presented more frequently than others, they eclipse the influence of the preceding trial. The Instance model of Logan (1988) provides a simple and elegant explanation by postulating a race between memory episodes. According to the Instance Theory, the attended stimulus and response features of each trial are stored in memory as a memory episode (or instance). Encoding a given task stimulus activates all stored memory episodes associated with this task and the fastest then determines the response. Transferred to our current findings, an according assumption is that if the stimuli are randomly presented as was the case in Experiment 1, all memory episodes are equally frequent, such that the most recent episode might have the highest chance to determine the response. However, changing the distribution of the memory episodes by presenting frequent SRTT–tone combinations, as we did in Experiment 2, increases the likelihood that one of these more frequent instances wins the retrieval race. We suspect that with training, this leads to a strong expectation about the current stimulus combination which becomes stronger than that of the *n* − 1 episode. This then reduces the partial-repetition costs.

On a more general level, this assumption would imply that presenting two tasks within a dual-tasking trial does not mean that the participants represent them as separate task-sets. Rather, it seems likely that they may conceptualize them as one single task-set (Schumacher & Hazeltine, 2016; Künzell et al., 2018). Our results suggest that this common task-set is further strengthened by storing the two processed tasks within one common memory episode. As a consequence, this results in partial-repetition costs in subsequent trials and probably also in a general confusion contributing to the costs typically obtained in dual-tasking (Liepelt et al., 2011; Strobach et al., 2014). Temporally postponing the processing of the secondary task might be one solution to hinder storing the two tasks in a common memory episode and in this way to cope with the resulting confusion between the two tasks (Logan & Gordon, 2001).

Furthermore, our findings of Experiment 3 suggest that probably the acquisition of such common memory episodes during training is not carved in stone. The results showed that due to the strong within-task coherence caused by the salient and easy-to-learn sequence built into the SRTT, the partial-repetition costs are reduced across training. We suspect that the built-in sequence might have contributed to a content-dependent separation of the two stimuli. One possibility is that the strength of the contingencies within the SRTT outperformed the formation strength of the contingencies across tasks within the dual-tasking trials, leading to separate memory episodes of the two tasks. For instance, findings in the field of statistical learning suggest that regularities built into a task can attract attentional selection (Kong et al., 2020; Zhao et al., 2013). Our current results are in line with these findings. However, more research is needed to further investigate this suggestion, as the built-in sequence might have simply allowed for advance response preparation which then reduced the stimulus-based interference. It is important to note that such an assumption and our episodic retrieval account are not mutually exclusive, because it would also imply that other memory episodes can eclipse the influence of the Trial *n* − 1 episodes. The fixed repeating sequence in the SRTT might lead to representing the SRTT stimuli more distinctly lowering the chance of erroneous instance retrieval due to partial repetition. For instance, according to chaining models of representation of serial order (e.g., Botvinick & Plaut, 2006; Wickelgren, 1969), the representation of an event (e.g., SRTT target in the middle) can be enriched by predecessor and successor information (e.g., the target in the middle which follows the target on the left vs. the middle target which follows the right target).

As a further point, it seems at first glance that our findings are at odds with recent results reported by Freedberg et al. (2014). The authors did not find task integration unless they explicitly instructed the participants to conceptualize the two tasks as belonging together (see also Schumacher & Hazeltine, 2016). However, there is a subtle, but important, methodological difference between the study of Freedberg et al. (2014) and our present experiments which might explain this disparity. Their training phase contained equal numbers of single- and dual-task blocks. From the perspective of the Instance Theory (Logan, 1988), it is thus conceivable that these single-task blocks have led to a large number of episodes representing stimulus and response of one task. Thus, different to the equal distribution of all task combinations in our Experiment 1, the Trial *n* − 1 episode might have had simply a smaller chance to win the race in the Freedberg et al.’s study. Yet, also here more research is needed to clarify the effects of single-task blocks on task integration in dual-tasking.

One limitation of our study considering further interpretation of the current results refers to the exclusion of the partial repetitions in the SRTT. Because we did not investigate partial repetitions on the first task, we are limited to looking into the effect of repetitions on the second task has on the first task. Although it would be interesting to look at the effect of repetitions on the first task has on the second task, this comparison was not possible for Experiment 2 due to the very few number of trials in the respective category. Since that made the comparison between Experiments 1 and 2 difficult, we refrained from analyzing this effect. With regard to the comparison between Experiments 1 and 3 looking into the effect of repetitions within SRTT would not be conclusive either, because in Experiment 3, we tested for an increase of within-task coherence. Findings from other single-task experiments with the SRTT indicate that response repetitions sometimes lead to slower responses rather than to the usually expected response time accelerations (Jiménez, 2008). It is conceivable that response repetitions in the SRTT might reflect inhibition processes due to the strong sequential character of this task.

One last question concerns the relation between our proposed episodic retrieval account and the debate in dual-tasking concerning parallel versus serial processing (Fischer & Plessow, 2015; Lehle et al., 2009; Miller et al. 2009; Navon & Miller, 2002). Probably, parallel processing in dual-tasking is best understood as task integration, as we try to propose here. However, there seems to be a crucial conceptual difference. Task integration, as it is understood here, results from the memory episodes of the processed trials. According to Logan’s (1988) Instance model, these episodes are automatically retrieved if the same information or parts of it re-appear. These memory episodes may, however, not automatically determine the responses, as was originally assumed by Logan (1988). Rather, it is conceivable that, in the sense of the prepared reflex (Hommel et al., 2001), they merely pre-activate the responses before the actual response is selected (Frings et al., 2020; Paelecke & Kunde, 2007). If this were true, the response–selection process itself can, despite of this pre-activation, occur either in a parallel or in a serial manner. As suggested here, the episodic retrieval hypothesis leads to task integration which might result in task confusions often solved by serial task processing (Koch et al., 2010; Logan & Gordon, 2001).

The findings of Zhao et al. (2020) might provide some support for the argument that the memory episodes only pre-activate the responses. They combined training in a four-element first-order SRTT with a second spatial task with random stimulus sequence. Due to the spatial feature overlap between the two tasks, they did not only obtain congruency effects between the two tasks, but found also that these congruency effects were modulated by the congruency effect four trials ago (i.e., one loop of the SRTT). That means, the RTs were shorter when the Trial *n* − 4 and Trial *n* were both congruent (C–C trials) than when the Trial *n* − 4 was congruent, but Trial n was incongruent (C-I trials, the typical Gratton-effect; Gratton et al., 1992). Thus, the authors provided evidence for the assumption that the two tasks of a trial are stored as a single integrated memory episode, which is retrieved if parts of this episode re-appear. Nevertheless, since they controlled carefully for the number of partial repetitions within the respective cells, the here proposed episodic memory retrieval cannot account for the found sequential modulation of the congruency effect. Rather, it seems that the response selection process itself led to the observed conflict (see also Janczyk, 2016). Such a finding might be better in line with the assumption that memory retrieval does not determine the response, but only pre-activates the response. Even this, however, is a question for further research.

## Conclusion

Overall, the current findings suggest that in dual-tasking experiments with short stimulus-onset asynchronies the participants often conceptualize the two tasks as belonging together. According to the results presented here, this leads to the development of memory episodes representing both tasks together, as opposed to separate memory episodes representing each task individually. This results in task confusions and as such contributes to dual-task costs.

## Data Availability

The software and the merged raw data are available at https://osf.io/maf9d/.

## Footnotes

1 In the SRT task, 0.09% of the trials were classified as RT outliers and 0.59% of the trials were classified as errors. In the tone discrimination task, 0.57% of the trials were classified as RT outliers. In 2.17% of the trials, the verbal response was incorrect and could not be correctly classified by the evaluation program in 5.09% of the cases. Due to exclusion of all trials with a PR of the SRTT-positions (16.82%) and every first trial of each block, overall 23.74% of all trials were excluded.

2 In principle, there are two different plausible baselines: full switches (FS) or full repetitions (FR). We decided to use FR rather than FS as the appropriate baseline to investigate the partial repetitions costs, because FR should reflect the re-activation of the memory episode of the preceding trial and we aimed at testing how retrieval of frequent stimulus combinations with practice eclipses this *n* − 1 effect. As we could have used trials with no competing trial-1 retrieval (i.e., FS-trials) as baseline, we checked whether our results depend upon the choice of the baseline. We conducted the same linear trends for all experiments also with FS as a baseline. In the SRTT, except for Experiment 3, we observed the same results as for the FR (Exp 1: *t*(25) = 0.65, *p* = 0.52, *d* = 0.13, Exp 2a: *t*(23) = 2.14, *p* < 0.05, *d* = 0.44, Exp 2b: *t*(24) = 1.99, *p* < 0.05, *d* = 0.4, Exp 3: *t*(24) = 0.18, *p* = .86, *d* = 0.04), In the tone-discrimination task, FR or FS as a baseline also did not change the pattern of results in Experiments 1 and 2a (Exp 1: *t*(25) = 0.66, *p* = 0.51, *d* = 0.13, Exp 2a: *t*(23) = 2.76, *p* < 0.01, *d* = 0.56). In Experiment 2b, the linear contrast just failed to achieve significance (Exp 2b: *t*(24) = 1.91, *p* = 0.06, *d* = 0.38). In Experiment 3, no significant effect was found at all (Exp 3: *t*(24) = 0.31, *p* = .76, *d* = 0.15). With the exception of Experiment 3, the patterns of results were quite similar for analyses using either FS or FR as a baseline. The difference between baselines in Experiment 3 might be due to the salient properties of the sequence structure. With learning, participants got faster for sequence position switches than repetitions. This might have attenuated the training-based reduction of the partial-repetition costs when using FS as baseline.

3 In the SRTT, 0.15/0.22% of the trials were classified as RT outliers and 0.6/0.46% of the trials were errors in Experiment 2a and Experiment 2b, respectively. In the tone discrimination task, 0.68/0.73% of the trials were classified as RT outliers. In 1.34/1.87% of the trials, the verbal response was incorrect, and in 1.09/3.02% of the cases, it could not be correctly classified by the evaluation program. Due to exclusion of all trials with a PR of the SRTT position (5,61/7,4%) and every first trial of each block, overall 9.4 and 13.5% of all trials were excluded in Experiments 2a and 2b, respectively.

4 In the SRT task, 0.73% of the trials were classified as RT outliers and 1.5% as errors. In the tone discrimination task, 0.18% of the trials were classified as RT outliers. In 3.27% of the trials, the verbal response was incorrect and in 9.6% of the cases not correctly classified by the evaluation program. Due to exclusion of all trials with a PR of the SRTT position (16.82%) and every first trial of each block, overall 29.49% of all trials were excluded.
